# Supplementary material for: Functional Analysis of Conserved Hypothetical Proteins from the Antarctic Bacterium, Pedobacter cryoconitis Strain BG5 Reveals Protein Cold Adaptation and Thermal Tolerance Strategies
Source: Microorganisms. 2022 Aug 16;10(8):1654. doi: 10.3390/microorganisms10081654 (PMC9415557; doi:10.3390/microorganisms10081654)
Supplement: Supplementary file 1 [file microorganisms-10-01654-s001.zip › microorganisms-1812106-supplementary.pdf]

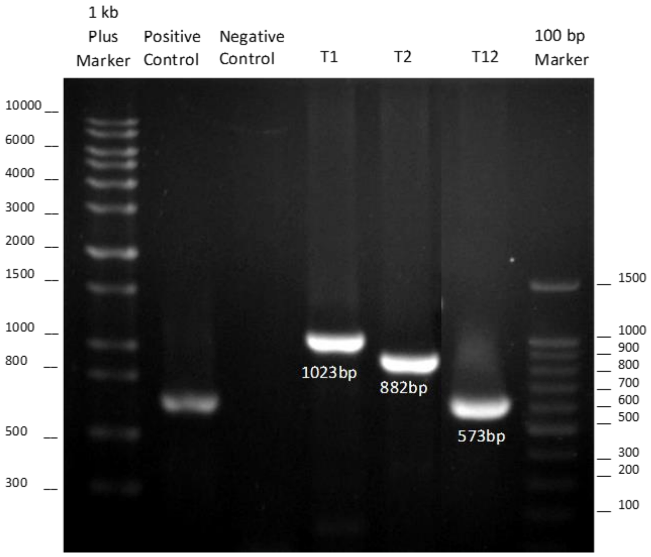

Figure S1. DNA amplification of *pcbg5hp1, pcbg5hp2,* and *pcbg5hp12* from *P. cryoconitis* BG5. The PCR products of *pcbg5hp1* (T1), *pcbg5hp2* (T2), and *pcbg5hp12* (T12) genes were indicated by the single bands on 1% agarose gel. 2nd

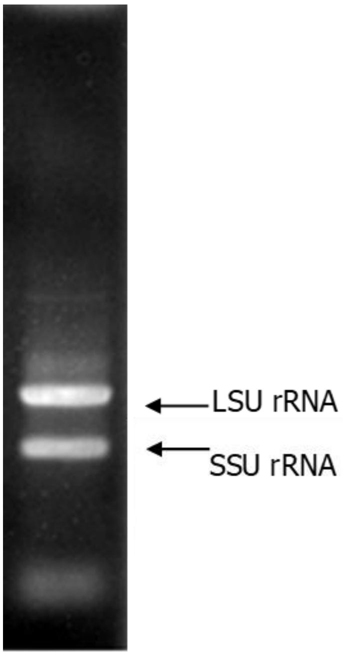

Figure S2. Agarose gel electrophoresis analysis of total RNA samples extracted from *P. cryoconitis*

Table S1. Primer design for PCR amplification of selected *P. cryoconitis* genes.

Gene	Direction	Primer DNA sequence	% GC content	Melting Temp
pcbg5hp1	Forward	5'- GGT GAT GAT GAT GAC AAG ATG GCT ACA TCA G-3'	45.2	60.2°C
	Reverse	5'- GGA GAT GGG AAG TCA TTA TCA AGA GAA GTC G-3'	45.2	59.6°C
pcbg5hp2	Forward	5'- GGT GAT GAT GAT GAC AAG ATG AAA ATT GCA ATA GC -3'	37.1	59.8°C
	Reverse	5'- GGA GAT GGG AAG TCA TTA AGA GAA AAA AGG TG -3'	40.6	58.9°C
pcbg5hp12	Forward	5'- GGT GAT GAT GAT GAC AAG ATG ACA ATC AAT AAC -3'	36.4	57.8°C
	Reverse	5'- GGA GAT GGG AAG TCA TTA CTA CTT CTT GTT ATT C -3'	38.2	58.1°C

Table S2. Gene's identification information of *P. cryoconitis* conserved HPs related to the thermal stress response.

Gene identifier	Gene identification number in the database	GenBank accession number
pcbg5hp1	NODE_14_length_965660_cov_19.618408	MT670404
pcbg5hp2	NODE_1_length_115730_cov_23.127287	MT670405
pcbg5hp12	NODE_14_length_965660_cov_19.618408	MT670415

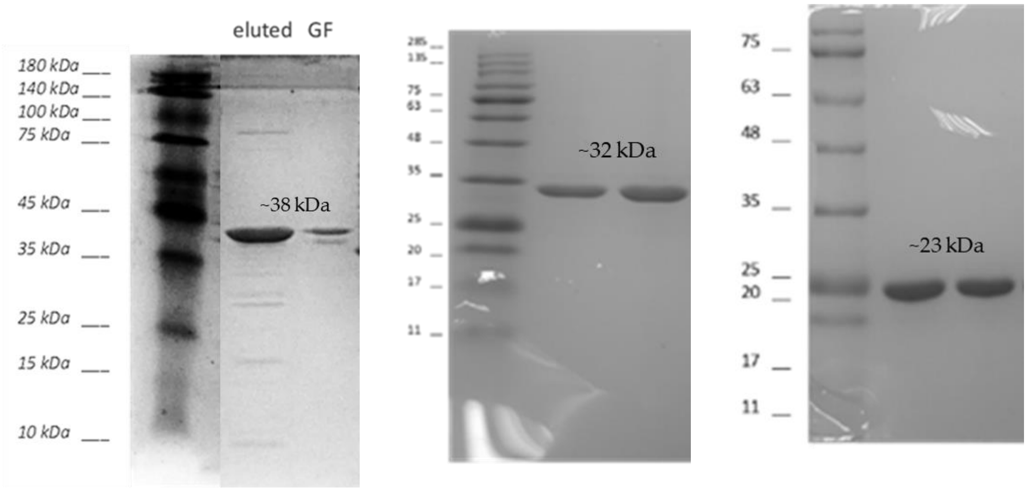

(a) PCBG5HP1 (b) PCBG5HP2 (c) PCBG5HP12

Figure S3. SDS-PAGE analysis of the purified protein of PCBG5HP1, PCBG5HP2, and PCBG5HP12 proteins from *P. cryoconitis*. (**a**) Purified PCBG5HP1 (~37 kDa) after Ni^{2+}-NTA affinity chromatography and Gel Filtration (**b**) Purified PCBG5HP2 (~32 kDa) after Gel Filtration. (**c**) Purified PCBG5HP12 (~23 kDa) after Gel Filtration.
